# *Ascodesmisrosicola* sp. nov. and *Talaromycesrosarhiza* sp. nov., two endophytes from *Rosaroxburghii* in China

**DOI:** 10.3897/BDJ.9.e70088

**Published:** 2021-12-23

**Authors:** Hong Zhang, Tian-Peng Wei, Yu-Tao Mao, Ming-Xia Ma, Kai Ma, Ying Shen, Mei-Juan Zheng, Wei-Yu Jia, Ming-Yan Luo, Yan Zeng, Yu-Lan Jiang, Guang-Can Tao

**Affiliations:** 1 Department of Plant Pathology, College of Agriculture, Guizhou University, Guiyang, China Department of Plant Pathology, College of Agriculture, Guizhou University Guiyang China; 2 Guizhou Academy of Testing and Analysis, Guiyang, China Guizhou Academy of Testing and Analysis Guiyang China; 3 Grain and Oil Quality Testing Center of Guiyang, Guiyang, China Grain and Oil Quality Testing Center of Guiyang Guiyang China

**Keywords:** multigene phylogenetics, morphology, new taxa, taxonomy, endophytic fungi

## Abstract

**Background:**

*Rosaroxburghii* Tratt., a deciduous shrub of the family Rosaceae, is usually used as food and medicinal materials and also cultivated as an ornamental. Plant endophytic fungi are a large class of microbial resources not fully researched, with great potential applications. Two strains of *Ascodesmis* and *Talaromyces* were isolated during a survey of biodiversity on endophytic fungi of *R.roxburghii* in China. Multigene phylogenetic analyses showed that each of the two fungi formed a distinct lineage and separated from known congeneric species and they are proposed as two novel taxa.

**New information:**

*Ascodesmisrosicola* sp. nov. usually has one or two conspicuous simple or branched ridges extending to the majority of the ascospore surface and remarkably small asci, distinguishing it from the previously-described species in the genus *Ascodesmis*. *Talaromycesrosarhiza* sp. nov., of the section Talaromyces, is closely related to *T.francoae*. It differs from the latter by having both monoverticillate and biverticillate conidiophores, while those of *T.francoae* are biverticillate. Both novel endophytes are illustrated and described.

## Introduction

*Rosaroxburghii* Tratt. is currently attracting significant attention for notably high vitamin C, superoxide dismutase and flavonoids (Xu et al. 2019). Vitamin C content of Gui Nong No. 5, one of the main cultivars, is 10–100 times higher than many other fruit and vegetable species, reaching 1000 mg/100 g (Xu et al. 2019). *Rosaroxburghii* is commonly used to treat diseases in traditional Chinese medicine, such as scurvy, type 2 diabetes mellitus and cacochylia (Wang et al. 2018, Wang et al. 2020). Its fruit is also widely applied in food and cosmetics for the development of functional products, due to diverse bioactive compounds with potential health-promoting properties (Yang et al. 2020). However, little is known about the endophytic fungi associated with this plant.

Endophytic fungi are phylogenetically diverse microorganisms that can colonise asymptomatically in various parts of living and healthy plants, ranging from the roots, stems and leaves to the flowers, fruits and seeds. To date, endophytes have been obtained from almost all plants investigated. Woody plants, in particular, may contain hundreds or thousands of fungal endophytes (Faeth and Fagan 2002, Jia et al. 2016). There are many endophytic fungi with various biological activities, such as growth promotion by producing plant hormones, enhancing host plant resistance to stresses through the production of bioactive compounds and facilitating the accumulation of medicinal ingredients, which is especially important for medicinal plants (Jia et al. 2016). Furthermore, they are also potential and inexhaustible bio-resources of many biologically-active compounds for medicinal applications (Abdel-Aziz et al. 2020). However, one should note that fungal endophytes may become pathogenic in the aging period of host plants or under specific environmental conditions (Park et al. 2017). Therefore, endophytic fungi play significant roles in agriculture and medicine.

Endophytic fungi belong to Ascomycota, Basidiomycota and Zygomycota, but Ascomycota is the predominant group compared to others (Gupta et al. 2020). During surveys of endophytic fungi associated with *R.roxburghii*, two strains of *Ascodesmis* and *Talaromyces* were collected in Guizhou Province (China). The genus *Ascodesmis* was introduced by Tieghem (1876), characterised by ascomata consisting of an unprotected bundle of asci only. *Ascodesmisaurea* and *A.nigricans* were considered to be the basic type of the Discomycetes (Brummelen 1981). Since 1981, no new species of *Ascodesmis* have been described worldwide. Ascospores are one of the most prominent morphologic features for species identification. Immature ascospores are hyaline and smooth before forming ornamentation. Upon maturity, the ascospores become pale to dark brown (Brummelen 1981, Kristiansen 2011).

The genus *Talaromyces*, within the order Eurotiales, was erected by Benjamin (1955) to accommodate teleomorphic *Penicillium* spp. The characteristic is soft cleistothecial ascomata, which are generally surrounded by multi-layered interwoven hyphae. This genus was redefined by Stolk and Samson (1972) and restricted to species producing only asci in chains. All gymnothecial ascomycetes that had the *Penicillium* state were included in *Talaromyces* by Pitt (1979). Later, phylogenetic research suggested that *Talaromyces* spp. and members of PenicilliumsubgenusBiverticillium belonged in a clade distinct from *Penicillium* sensu stricto (Houbraken and Samson 2011, Chen et al. 2016). Thereafter, all PenicilliumsubgenusBiverticillium spp. were transferred to *Talaromyces* according to the principle of nomenclatural priority and single name nomenclature (Samson et al. 2011). Subsequently, *Talaromyces* was subdivided into seven sections with eighty-eight species, based on multi-gene phylogenetic analyses, combined with morphological observations. These sections were *Bacillispori*, *Helici*, *Islandici*, *Purpurei*, *Subinflati*, *Talaromyces* and *Trachyspermi* (Yilmaz et al. 2014, Chen et al. 2016). Lately, a new section, section Tenues, was introduced by Sun et al. (2020) for *T.tenuis*. To date, this genus consists of more than 180 species, classified into eight sections.

In this study, we introduce *Ascodesmisrosicola* and *Talaromycesrosarhiza* as two novel taxa. Both of them are confirmed by multigene phylogeny and morphological characters.

## Materials and methods

### Sample collection

The wild-type *R.roxburghii* was collected from Guizhou Province, China (April and August 2020) (Table 1). Healthy tissues (roots and fruit) of *R.roxburghii* were collected randomly from the different sampling sites. All materials were sent to the laboratory immediately and stored in a refrigerator at 4°C. Each sample tissue was examined within 48 hours of collection (Ranjan et al. 2019).

### Isolation and culture

All tissues, especially roots, were cleaned under tap water for half an hour, rinsed with double-distilled water for 10 min，and dried under natural conditions. Then, they were cut into small pieces and transferred to the clean bench for surface disinfection. The margin of these pieces was trimmed off under aseptic conditions. All samples were surface-sterilised in 75% ethanol (1 min) and rinsed three times with sterile water. Subsequently, they were treated with 1% (w/v) aqueous sodium hyprochlorite (NaOCl) for several minutes (roots, 2 min; fruit, 1 min) and washed three times with sterile water again. After washing, the surface water of tissues was blotted with sterile filter papers. The potato dextrose agar (PDA, Shanghai Bio-way Technology Co., Ltd., China) medium had been supplemented with streptomycin sulphate (0.5 g/l) to avoid bacterial contamination. Each culture plate contained three segments. These plates were incubated at 28±1°C in a 12-h light:12-h dark photoperiod for 3 to 7 days. After several days, hyphal tips were subcultured into fresh PDA plates to obtain pure endophytic strains (Ranjan et al. 2019).

The effectiveness of surface sterilisation was monitored with three methods. First, the final rinsing water (0.1 ml) was coated on PDA plates (Rojas et al. 2020). The second way was that the surface-sterilised plant materials were rolled for 1 min and placed for 20 min on PDA plates and then removed (Li et al. 2016, Singh et al. 2017). In addition, during the isolation process, three open PDA plates were placed in the clean bench to confirm that colonies growing on isolated plates were produced from plant tissues and not contaminating fungi from the environment.

### Morphology

Morphological identification was performed with PDA (Shanghai Bio-way Technology Co., Ltd., China), oatmeal agar (OA, Beijing Solarbio Science & Technology Co., Ltd., China), and malt-extract agar (MEA, Shanghai Bio-way Technology Co., Ltd., China). Macroscopic morphology, for example, growth rate, colony colour and pigmentation, were examined at 2-day intervals. Microscopic characteristics of ascospores or conidia were determined and recorded and spores of each strain were randomly selected for measurement (length and width) (Quezado et al. 2010), with the range and mean calculated. The colonies were observed under a stereomicroscope and morphological characters were confirmed by optical microscope (Olympus BX53, Japan).

### DNA extraction, PCR amplification and sequencing

DNA was extracted from fresh mycelia grown on PDA for 7 to 10 days using the Fungal gDNA Isolation Kit (BW-GD2416, Biomiga, China), following the manufacturer’s instructions. The products were stored at -20°C until polymerase chain reaction (PCR) was performed. PCR amplifications were conducted on a T100 Thermal Cycler (BIO-RAD, USA). According to manufacturer’s instructions in a total volume of 25 μLl, including 12.5 µl 2X SanTaq PCR Mix (the mixture of MgCl_2_, dNTP, Taq DNA Polymerase, PCR buffer and PCR enhancer solution), 9.5 µl double-distilled water, 1.0 µl each of forward and reverse primers (0.05–1 µmol/l) and 1.0 µl DNA sample (1–10 ng/µl). The forward and reverse PCR reaction primers are shown in Table 2. The ITS, LSU, BenA, CaM and RPB2 genes were amplified as previously mentioned by White et al. (1990), Vilgalys and Hester (1990), Glass and Donaldson (1995), Hong et al. (2006) and Liu et al. (1999). The amplified PCR products were directed to Sangon Biotech (Shanghai) Co., Ltd. (Shanghai, P. R. China), a commercial sequencing provider.

### Phylogenetic analyses

Forward and reverse sequences were utilised to create consensus sequences by BioEdit v. 7.0.9.0 (Hall 1999) and BLASTn searched in NCBI (https://blast.ncbi.nlm.nih.gov/Blast.cgi) to identify the genus-level taxonomic status. Phylogenetic analyses were performed, based on the sequences generated in this study and recently published data (Hansen et al. 2013, Vu et al. 2019, Schoch et al. 2009, Hansen et al. 2005, Lindemann et al. 2019, Chen et al. 2016, Manoch et al. 2013, Sun et al. 2020, Visagie et al. 2015, Wang et al. 2016, Wang et al. 2017, Yilmaz et al. 2012, Yilmaz et al. 2014, Yilmaz et al. 2016), which were downloaded from GenBank (https://www.ncbi.nlm.nih.gov/genbank/) (Table 3). The multiple alignments were automatically generated using MAFFT v. 7 webserver (https://mafft.cbrc.jp/alignment/server/) (Katoh et al. 2019). Alignments were also adjusted manually when needed in BioEdit v. 7.0.9.0 (Hall 1999) and concatenated in PhyloSuite v. 1.2.2 (Zhang et al. 2020).

Maximum Likelihood (ML) and Bayesian Inference (BI) methods were used to construct the phylogenetic trees. The best-fit partition models were inferred for the independent sequence datasets using ModelFinder (Kalyaanamoorthy et al. 2017) integrated into PhyloSuite (Zhang et al. 2020) and the results were used for ML and BI (Table 2). ML phylogenies were conducted using IQ-TREE (Nguyen et al. 2014) under the edge-linked partition model for 10,000 ultrafast (Minh et al. 2013) bootstraps. Bootstrap support (BS) values were evaluated with 1000 replicates. BI phylogenies were calculated using MrBayes 3.2.6 (Ronquist et al. 2012) under the partition model (2 parallel runs, 2,000,000 generations), sampling every 1000 generations, the initial 25% of sampled data were discarded as burn-in and the remaining samples were used to generate the majority consensus tree and estimate the posterior probabilities (PP) (Maharachchikumbura et al. 2015). Phylograms were visualised in FigTree v.1.4.3 (Rambaut 2014) with bootstrap values (BS/PP) above or below the nodes and reorganised in Adobe Illustrator CC 2019.

## Taxon treatments

### Ascodesmis rosicola

H. Zhang & Y. L. Jiang sp. nov.

84EB7587-06C3-5B57-AEB1-4F6A365B8487

IF556900

#### Materials

- **Type status:** Holotype. **Occurrence:** catalogNumber: GUCC 190035.1; recordedBy: Hong Zhang; **Taxon:** scientificName: *Ascodesmisrosicola*; kingdom: Fungi; phylum: Ascomycota; class: Pezizomycetes; order: Pezizales; family: Ascodesmidaceae; genus: Ascodesmis; **Location:** country: China; stateProvince: Guizhou; locality: Guiyang; **Identification:** identifiedBy: Hong Zhang; dateIdentified: 2021
- **Type status:** Other material. **Occurrence:** catalogNumber: GUCC 190204.1; recordedBy: Hong Zhang; **Taxon:** scientificName: *Ascodesmisrosicola*; kingdom: Fungi; phylum: Ascomycota; class: Pezizomycetes; order: Pezizales; family: Ascodesmidaceae; genus: Ascodesmis; **Location:** country: China; stateProvince: Guizhou; locality: Guiyang; **Identification:** identifiedBy: Hong Zhang; dateIdentified: 2021

#### Description

**Figure 1.**
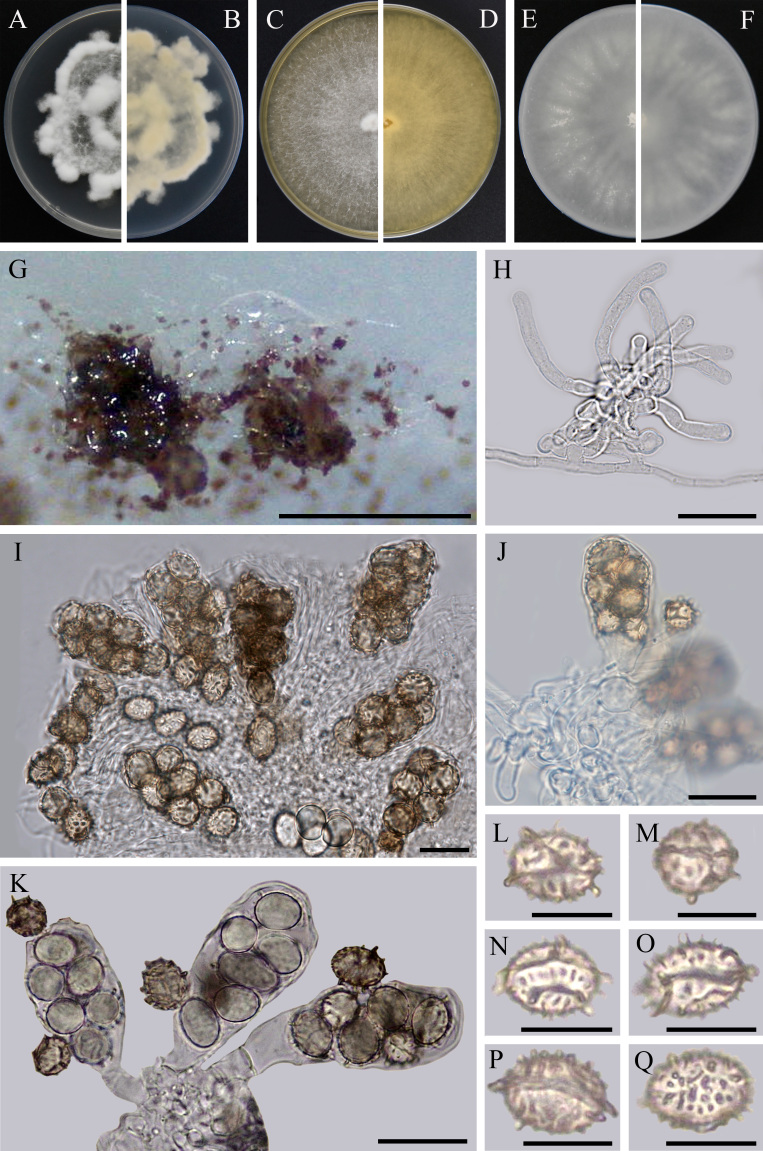
*Ascodesmisrosicola* (GUCC 190035.1, holotype). **A-F** On PDA, MEA and OA, respectively in 14 days at 28°C (**A**, **C** and **E** from above, **B**, **D** and **F** from below); **G** Sexual morph on SNA; **H** Paraphyses; **I-K** Apothecium and asci; **L-Q** Ascospores. Scale bars: **G** = 0.5 mm, **H-K** = 20 μm, **L-Q** = 10 μm.

Endophytic fungi of *R.roxburghii*. **Asexual morph** not observed. **Sexual morph (Fig. 1)**: Apothecia gregarious, superficial, sessile, 200–320 µm diameter, first hyaline, becoming brownish upon maturity. Excipulum absent. Asci broadly clavate or obovoid, with a short broad stalk or a broad base, 30.5–50.0 × 12.5–21.0 µm (av. = 38.0 × 19.0 µm, n = 30), 6–8-spored. Ascospores irregularly arranged, ellipsoid to perfectly spherical (length/breadth ratio 1.01–1.69, av. 1.32), at first hyaline, brownish upon maturity, 5.0–13.0 × 4.5–10.0 µm (av. = 10.5 × 8.0 µm, n = 30), at first smooth, later ornamented with a very variable pattern of isolated warts and spines, occasionally with a few fine connectives and one or two very obvious simple or branched ridges extending over the major part of the ascospore surface, very few without ridge. Paraphyses septate, simple and hyaline, 3.5–6.5 µm thick.

Cultural characteristics: Description based on GUCC 190035.1. On PDA, reaching 6.0–7.5 cm in diameter after 14 days of cultivation in dark at 28°C, superficial, margin irregular, white, with abundant aerial mycelium, uneven, centre and margin uplifted; reverse yellowish. On MEA, 28°C: medium sparse, circular, flat. Mycelium white, cottony, margin regular. On OA, 28°C: radial, aerial mycelium sparse, pale white, growth regular. On MEA and OA, reaching 85 mm in diameter under the same conditions.

#### Etymology

The name refers to the host plant, *Rosaroxburghii*, from which this fungus was isolated.

#### Notes

**Figure 2.**
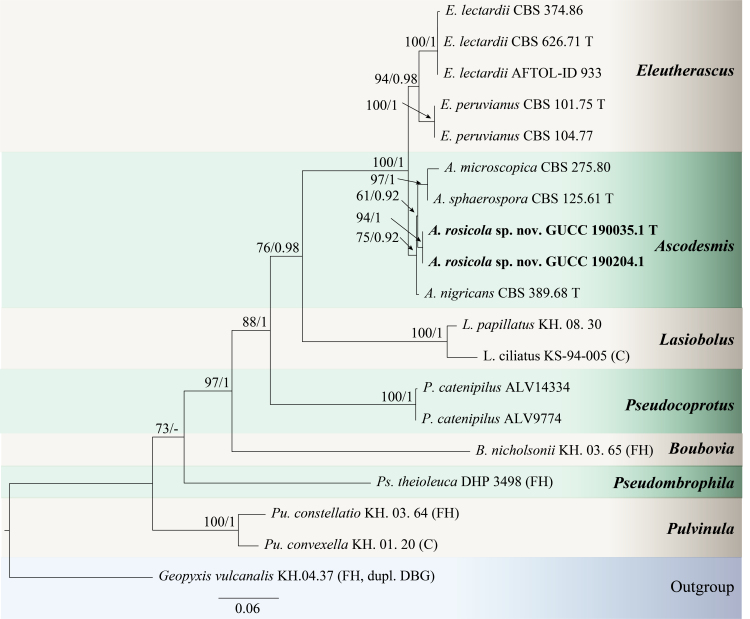
Phylogram generated from Maximum Likelihood analysis, based on combined ITS, LSU and RPB2 sequence data. *Geopyxisvulcanalis* (KH.04.37 (FH, dupl. DBG)) is used as the outgroup taxa. Bootstrap support values for ML greater than 50% and Bayesian posterior probabilities greater than 0.90 are given near nodes (BS/PP), respectively. The novel taxon is indicated in bold and black. T: type or ex-type.

*Ascodesmisrosicola* is introduced as a new species, based on morphological characteristic and phylogenetic analysis. The genus *Ascodesmis*, established by Tieghem (1876), presently contains 13 species (Trivedi et al. 1973, Brummelen 1981, Currah 1986), but these species have no available sequence data, except *A.microscopica*, *A.nigricans* and *A.sphaerospora*. In the phylogenetic analyses (Fig. 2), using the combined ITS, LSU and RPB2 sequence data of Pezizales, *A.rosicola* is related to *Ascodesmis* spp. The two *A.rosicola* isolates clustered with good support (BS = 94, PP = 1) and placed in a distinct clade, albeit with moderate support (BS = 61, PP = 0.92). This species shows some similarity to *A.porcina* in having ascospores with a conspicuously simple or branched ridge extending over the most part of the spore surface, which is well separated from other *Ascodesmis* spp. However, *A.rosicola* differs from *A.porcin* by its smaller asci (30.5–50.0 × 12.5–21.0 µm vs. 65–80 (–90) × 20–30 (–35) µm) and smooth surface of young ascospores (those of *A.porcin* are ornamented) (Brummelen 1981). Moreover, the hosts of *A.rosicola* and *A.porcin* (from the dung of donkey, goat, peccary, pig and rat) (Brummelen 1981) were notably different.

### Talaromyces rosarhiza

H. Zhang & Y. L. Jiang sp. nov.

3D225F1A-55E7-5704-897A-D2ACAF5C22F0

IF556981

#### Materials

- **Type status:** Holotype. **Occurrence:** catalogNumber: GUCC 190040.1; recordedBy: Hong Zhang; **Taxon:** scientificName: *Talaromycesrosarhiza*; kingdom: Fungi; phylum: Ascomycota; class: Eurotiomycetes; order: Eurotiales; family: Trichocomaceae; genus: Talaromyces; **Location:** country: China; stateProvince: Guizhou; locality: Guiyang; **Identification:** identifiedBy: Hong Zhang; dateIdentified: 2021
- **Type status:** Other material. **Occurrence:** catalogNumber: GUCC 197011.1; recordedBy: Hong Zhang; **Taxon:** scientificName: *Talaromycesrosarhiza*; kingdom: Fungi; phylum: Ascomycota; class: Eurotiomycetes; order: Eurotiales; family: Trichocomaceae; genus: Talaromyces; **Location:** country: China; stateProvince: Guizhou; locality: Guiyang; **Identification:** identifiedBy: Hong Zhang; dateIdentified: 2021

#### Description

**Figure 3.**
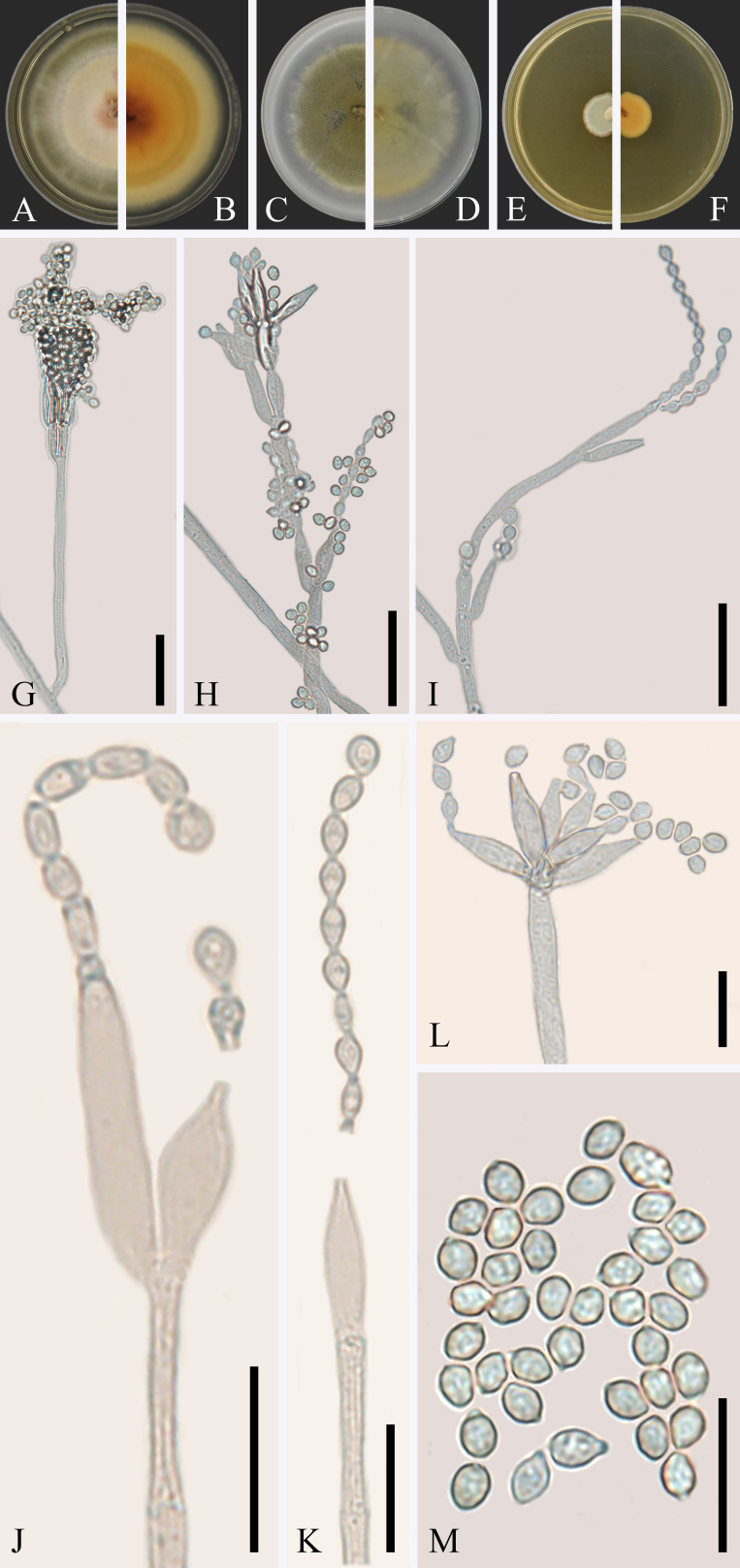
*Talaromycesrosarhiza* (GUCC 190040.1, holotype). **A-F** On PDA, OA and MEA, respectively in 14 days at 28°C (**A**, **C** and **E** from above; **B**, **D** and **F** from below); **G-M** Asexual morph on OA (**G-L** Conidiophores; **M** Conidia). Scale bars: **G-I** = 20 μm, **J-M** = 10 μm.

Endophyte of *R.roxburghii*. **Sexual morph** not observed. **Asexual morph** (Fig. 3): Mycelium superficial, pale brown, septate, branched. Conidiophores monoverticillate and biverticillate, a minor proportion terverticillate, macronematous, mostly straight, smooth, branched, thick-walled. For biverticillate conidiophores, metulae 2–3, appressed or divergent, 6.0–14.5 × 1.5–3.0 µm (av. = 10.5 × 2.5 µm, n = 30); phialides ampulliform, tapering into very thin neck, 2–3 per metula, 6.5–15.0 × 1.5–3.5 µm (av. = 10.0 × 2.5 µm, n = 30). For monoverticillate ones, phialides 1–6, ampulliform, tapering into very thin neck, 10.5–16.0 × 2.5–4.0 µm (av. = 12.5 × 3.0 µm, n = 30), conidia subglobose to ellipsoidal, smooth-walled, 2.5–4.0 × 2.0–3.0 µm (av. = 3.0 × 2.5 µm, n = 30).

Cultural characteristics: Description based on GUCC 190040.1. On PDA, reaching 50 mm in diameter after 14 days of cultivation in dark at 28°C; moderately deep, slightly sulcate, flat; margin entire, mycelium white. On OA, reaching 42 mm in diameter under the same conditions, low, flat; margin low, entire; mycelium white; velvety; abundant sporulation; conidia en masse dark olive green. On MEA 28°C, 14 days: reaching 12 mm; raised; margin low, flat, entire; mycelium white; floccose to velvety; abundant sporulation, conidia en masse dull green; soluble pigments absent; exudates absent; reverse greyish orange.

#### Etymology

The word "rosarhiza" originated from “rosa” referring to the host plant, *Rosaroxburghii* and “rhiza” referring to root, from which this fungus was isolated.

#### Notes

**Figure 4.**
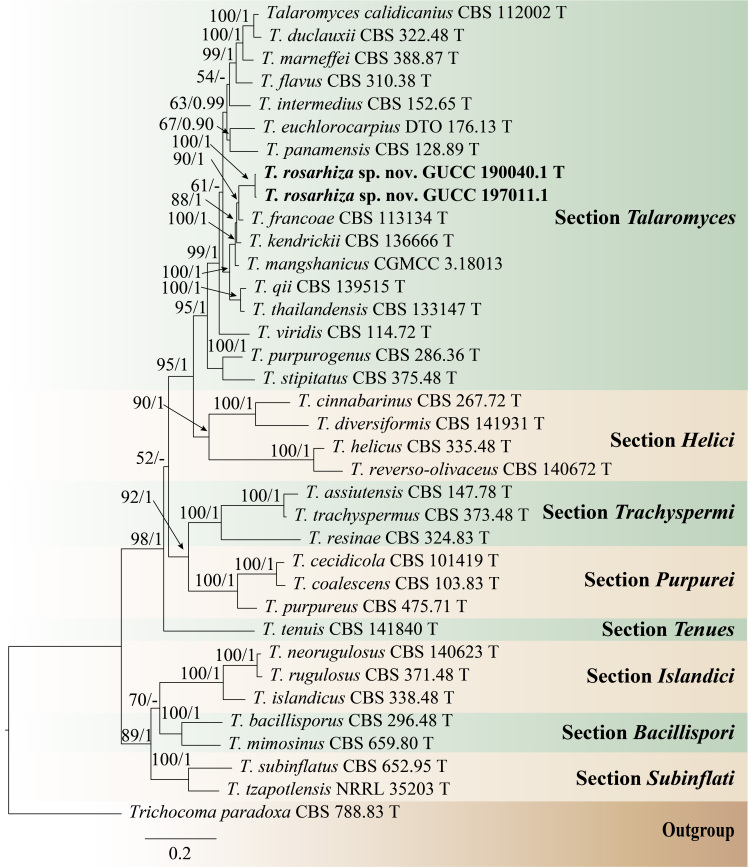
Phylogram generated from Maximum Likelihood analysis, based on combined ITS, BenA, CaM and RPB2 sequence data. *Trichocomaparadoxa* (CBS 788.83) is used as the outgroup taxa. Bootstrap support values for ML greater than 50% and Bayesian posterior probabilities greater than 0.90 are given near nodes (BS/PP), respectively. The new taxon is indicated in bold and black. T: type or ex-type.

*Talaromycesrosarhiza* is described as a new species, based on morphology and phylogenetic analyses. Phylogenetic analysis (Fig. 4) was carried out using combined ITS, BenA, CaM and RPB2 sequence data. Clustered together with *T.francoae*, *T.kendrickii*, *T.mangshanicus*, *T.qii* and *T.thailandensis* and belonged to section Talaromyces. The two *T.rosarhiza* isolates clustered with strong support (BS = 100, PP = 1) and closely related to *T.francoae*, but located in a distinct clade with good support (BS = 90, PP = 1). Detailed morphological differences between *T.rosarhiza* and its related taxa are summarised in Table 4 (Yilmaz et al. 2016, Visagie et al. 2015, Wang et al. 2017, Wang et al. 2016, Manoch et al. 2013). *Talaromycesrosarhiza* can be distinguished from *T.francoae* by its monoverticillate and biverticillate conidiophores (while *T.francoae* biverticillate), number of metulae per verticil (2–3 vs. 3–6), number of phialides per metulae (2–3 vs. 3–6), conidia shape (ellipsoidal vs. globose) and conidial wall (smooth vs. verrucose, rough) (Yilmaz et al. 2016).

## Analysis

### Phylogenetic analyses

***Ascodesmisrosicola***: For the genus *Ascodesmis*, only three species, *A.microscopica*, *A.nigricans* and *A.sphaerospora*, have available sequence data. Therefore, phylogenetic relationships were deduced using sequence data of Pezizales. Nineteen strains were included in the combined analyses (ITS, LSU and RPB2) which comprised 1999 characters (561 for ITS, 819 for LSU, 619 for RPB2). The tree topology of the ML analysis was similar to the BI analysis. Two new strains, GUCC 190035.1 and GUCC 190204.1, were related to *Ascodesmis* spp., clustered with good support (BS = 94, PP = 1) and formed an independent moderately-supported clade (BS = 61, PP = 0.92) (Fig. 2).

***Talaromycesrosarhiza***: Phylogenetic relationships were investigated using sequence data of *Talaromyces*. Thirty-six strains were included in the combined analyses (ITS, BenA, CaM and RPB2) which comprised 2549 characters (541 for ITS, 570 for BenA, 647 for CaM, 791 for RPB2). The tree topology of ML analysis was similar to the BI analysis. Two new strains, GUCC 190040.1 and GUCC 197011.1, clustered with strong support (BS = 100, PP = 1) and formed a group with five species of Talaromyces that had been reported and belonged to section Talaromyces. *Talaromycesrosarhiza* was closely related to *T.francoae*, but placed in a distinct clade with high bootstrap support (BS = 90) and posterior probability (PP = 1) (Fig. 4).

## Discussion

In this study, *A.rosicola* and *T.rosarhiza*, two new endophytes isolated from *R.roxburghii* in China, are proposed and described. Multigene phylogenetic analyses revealed that *A.rosicola* was phylogenetically close to *A.nigricans* and *A.sphaerospora* and was placed in a distinct clade with moderate support. However, combined with morphological characters, the novel species is confirmed. Similarly, based on morphology and phylogenetic analyses, *T.rosarhiza* is suggested as a new species.

Although there are 13 species of *Ascodesmis* listed in Mycobank (https://www.mycobank.org/), *A.aurea* and *A.hawaiiensis* are considered dubious species due to the lack of adequate description and material (Brummelen 1981). In addition, *A.caninus* and *A.reticulata* are two synonyms of *A.microscopica* and *A.echinulata* is regarded as a synonym of *A.nigricans* (*Brummelen 1981*). They are rarely collected, possibly because of their size, which hardly exceeds 0.5 mm in diameter or they are really rare (Kristiansen 2011).

*Talaromyces*, another genus in this study, is important in biotechnology, medicine and the food industry (Yilmaz et al. 2014). *Talaromyces* spp. are isolated from assorted substrates around the world (Yilmaz et al. 2016), including soil, plant, air, animals, food, dust, human and dung. Notably, many pathogenic fungi of *Talaromyces* have been shown to possess potential biological activities. For example, *T.albobiverticillius* causes post-harvest fruit rot on pomegranate (Mincuzzi et al. 2017)，but it has been shown to possess anti-inflammatory properties (Bai et al. 2020). *Talaromycespinophilus*, as a pathogen, has been reported to cause post-harvest rot of sugar beet (*Beta vulgaris*) (Haque and Parvin 2020); however, this fungus is also a promising biocontrol agent, able to inhibit *Pythium* and *Rhizoctonia*-induced damping-off of cucumber (Kazerooni et al. 2019). Thus, further investigations on the pathogenicity and biological activity of *T.rosarhiza* are needed.

In the present study, a total of 127 strains of endophytic fungi were successfully isolated from *R.roxburghii* and categorized into six classes (Sordariomycetes, Dothideomycetes, Eurotiomycetes, Pezizomycetes, Leotiomycetes and Agaricomycetes), of which strains GUCC 190035.1, GUCC 190204.1, GUCC 190040.1 and GUCC 197011.1 were identified as two new taxa. As known, besides agriculture and medicine, many endophytic fungi may play significant roles in shaping and maintaining the balance of microbial communities in plants (Su et al. 2016). Therefore, further research is also necessary for the ecological significance of *A.rosicola* and *T.rosarhiza*.

## Supplementary Material

XML Treatment for Ascodesmis rosicola

XML Treatment for Talaromyces rosarhiza

## Figures and Tables

**Table 1.** Location charateristics and climate features of samples collection sites

**Sampling site**	**Latitude and longitude**	**Altitude** （m）	**Mean annual temperature** （℃）	**Mean annual rainfall** （mm）	**Mean annual sunshine hours** （h）	**Sampling time**
Guiyang City, Guizhou Province	27°4'50"N, 106°29'50"E	1184	15.3	1130	1235	22 April 2020
Liupanshui City, Guizhou Province	25°52'52"N, 104°33'59"E	2047	15.2	1390	1593	4 August 2020

**Table 2.** The best-fit evolutionary models in the phylogenetic analyses.

**Genus**	**Phylogenetic analysis**	**Model**				
		**ITS (ITS4/ITS5)**	**LSU** **(LR0R/LR5)**	**BenA** **(Bt2a/Bt2b)**	**CaM** **(Cmd5/Cmd6)**	**RPB2** **(fRPB2-5F**/ **fRPB2-7cR)**
*Ascodesmis*	ML analysis	TNe+I+G4	TIM3+F+I+G4	-	-	TNe+I+G4
	BI analysis	GTR+F+I	GTR+F+I+G4	-	-	SYM+I+G4
*Talaromyces*	ML analysis	TNe+R3	-	TIM2e+I+G4	K2P+I+G4	K2P+I+G4
	BI analysis	GTR+F+I+G4	-	GTR+F+I+G4	SYM+I+G4	SYM+I+G4

**Table 3.** Taxa used in this study and their corresponding GenBank accession numbers.

**Species**	**Strain no.**	**GenBank accession no.**				
		**ITS**	**LSU**	**BenA**	**CaM**	**RPB2**
*Ascodesmismicroscopica*	CBS 275.80	MH861263	MH873032	-	-	-
*A.nigricans*	CBS 389.68 T	-	DQ168335	-	-	JX943761
*A.sphaerospora*	CBS 125.61 T	MH857994	MH869550	-	-	-
***A.rosicola* sp. nov.**	**GUCC 190035.1 T**	**MZ221601**	**MZ221605**	-	-	**MZ333139**
***A.rosicola* sp. nov.**	**GUCC 190204.1**	**MZ221602**	**MZ221606**	-	-	**MZ333140**
*Boubovianicholsonii*	KH.03.65 (FH)	-	DQ220395	-	-	JX943755
*Eleutherascuslectardii*	CBS 626.71 T	MH860289	MH872042	-	-	-
*E.lectardii*	AFTOL-ID 933	-	DQ470966	-	-	DQ470918
*E.peruvianus*	CBS 101.75 T	-	DQ220330	-	-	JX943760
*E.peruvianus*	CBS 104.77	MH861030	MH872802	-	-	-
*Geopyxisvulcanalis*	KH.04.37 (FH, dupl. DBG)	-	KC012680	-	-	JX943770
*Lasiobolusciliatus*	KS-94-005 (C)	-	DQ167411	-	-	-
*L.papillatus*	KH.08.30	-	KC012687	-	-	JX943758
*Pseudocoprotuscatenipilus*	ALV9774	MH846260	MH846258	-	-	MH844626
*P.catenipilus*	ALV14334	MH846261	MH846259	-	-	-
*Pseudombrophilatheioleuca*	DHP 3498 (FH)	-	KC012696	-	-	JX943756
*Pulvinulaconstellatio*	KH.03.64 (FH)	-	DQ062987	-	-	JX943773
*Pu.convexella*	KH.01.20 (C)	-	DQ062986	-	-	JX943772
*Talaromycesassiutensis*	CBS 147.78 T	JN899323	-	KJ865720	KJ885260	KM023305
*T.bacillisporus*	CBS 296.48 T= IMI 040045 = NRRL 1025	KM066182	-	AY753368	KJ885262	JF417425
*T.calidicanius*	CBS 112002 T	JN899319	-	HQ156944	KF741934	KM023311
*T.cecidicola*	CBS 101419 T= DAOM 233329	AY787844	-	FJ753295	KJ885287	KM023309
*T.cinnabarinus*	CBS 267.72 T= NHL 2673	JN899376	-	AY753377	KJ885256	JN121477
*T.coalescens*	CBS 103.83 T	JN899366	-	JX091390	KJ885267	KM023277
*T.diversiformis*	CBS 141931 T= CGMCC3.18204 = DTO 317-E3	KX961215	-	KX961216	KX961259	KX961274
*T.duclauxii*	CBS 322.48 T= IMI 040044 = MUCL 28672 = NRRL 1030	JN899342	-	JX091384	KF741955	JN121491
*T.euchlorocarpius*	PF 1203 = DTO 176-I3 T= DTO 176-I4	AB176617	-	KJ865733	KJ885271	KM023303
*T.flavus*	CBS 310.38 T= IMI 197477 = NRRL 2098	JN899360	-	JX494302	KF741949	JF417426
*T.francoae*	CBS 113134 T= IBT 23221 = DTO 056-D9	KX011510	-	KX011489	KX011501	MN969188
*T.helicus*	CBS 335.48 T= DSM 3705 = IMI 040593 = NRRL 2106	JN899359	-	KJ865725	KJ885289	KM023273
*T.intermedius*	CBS 152.65 T= BDUN 267 = IFO 31752 = IMI 100874	JN899332	-	JX091387	KJ885290	KX961282
*T.islandicus*	CBS 338.48 T= IMI 040042 = MUCL 31324 = NRRL 1036	KF984885	-	KF984655	KF984780	KF985018
*T.kendrickii*	CBS 136666 T = DTO 273-F4 = IBT 13593	KF741987	-	KF741921	KF741967	MN969158
*T.mangshanicus*	CGMCC 3.18013	KX447531	-	KX447530	KX447528	KX447527
*T.marneffei*	CBS 388.87 T = ATCC 18224= CBS 334.59 = IMI 068794ii = IMI 068794iii	JN899344	-	JX091389	KF741958	KM023283
*T.mimosinus*	CBS 659.80 T = FRR 1875 = IMI 223991	JN899338	-	KJ865726	KJ885272	MN969149
*T.neorugulosus*	CBS 140623 T= CGMCC3.18215 = DTO 318-A8	KU866659	-	KU866846	KU866743	KU867003
*T.panamensis*	CBS 128.89 T= IMI 297546	JN899362	-	HQ156948	KF741936	KM023284
*T.purpureus*	CBS 475.71 T= FRR 1731 = IMI 181546	JN899328	-	GU385739	KJ885292	JN121522
*T.purpurogenus*	CBS 286.36 T= IMI 091926	JN899372	-	JX315639	KF741947	JX315709
*T.qii*	AS3.15414 T= CBS 139515	KP765384	-	KP765380	KP765382	MN969164
*T.resinae*	AS 3.4387 = CBS 324.83 T= DTO 027-G5	MT079858	-	MN969442	MT066184	MN969221
*T.reverso-olivaceus*	CBS 140672 T= CGMCC3.18195 = DTO 317-C3	KU866646	-	KU866834	KU866730	KU866990
***T.rosarhiza* sp. nov.**	**GUCC 190040.1 T**	**MZ221603**	-	**MZ333143**	**MZ333137**	**MZ333141**
***T.rosarhiza* sp. nov.**	**GUCC 197011.1**	**MZ221604**	-	**MZ333144**	**MZ333138**	**MZ33314**
*T.rugulosus*	CBS 371.48 T= IMI 040041 = MUCL 31201 = NRRL 1045	KF984834	-	KF984575	KF984702	KF984925
*T.stipitatus*	CBS 375.48 T= NRRL 1006 = IMI 39805	JN899348	-	KM111288	KF741957	KM023280
*T.subinflatus*	CBS 652.95 T= IBT 17520	JN899397	-	KJ865737	KJ885280	KM023308
*T.tenuis*	CBS 141840 T = DTO 340-G9	MN864275	-	MN863344	MN863321	MN863333
*T.thailandensis*	CBS 133147 T= KUFC 3399	JX898041	-	JX494294	KF741940	KM023307
*T.trachyspermus*	CBS 373.48 T = IMI 040043	JN899354	-	KF114803	KJ885281	JF417432
*T.tzapotlensis*	NRRL 35203 T	KX946902	-	KX946884	KX946893	KX946922
*T.viridis*	CBS 114.72 T= ATCC 22467 = NRRL 5575	AF285782	-	JX494310	KF741935	JN121430
*Trichocomaparadoxa*	CBS 247.57, CBS 103.73, CBS 788.83 T	MH860643	-	JF417469	JF417506	JN121417

New species are marked in **bold**; T: indicates type or ex-type strains.

**Table 4.** Morphological comparisons between *Talaromycesrosarhiza* and its allies.

Species	Conidiophores pattern	No. of metulae per verticil	Metulae size (μm)	No. of phialides per metulae	Phialides size (μm)	Conidia shape	Conidial walls	Conidia size (μm)
***T.rosarhiza* sp. nov.**	Monoverticillate and biverticillate	2–3	6.0–14.5 × 2.0–3.0	2-3	6.5–15.0 × 1.5–3.5	Ellipsoidal	Smooth	2.5–3.5 × 2.0 –3.0
*T.francoae*	Biverticillate	3-6	8–13 × 2.5–4.5	3–6	8.5–12 × 2.5–4	Globose	Verrucose, rough	2.5–4 × 2.5–4
*T.kendrickii*	Biverticillate	3–8	10–13 × 3–4	3–5	9–12 × 2.5– 3.5	Subglobose	Roughened	2.5–3 × 2.5–3
*T.mangshanicus*	Biverticillate	3–6	11–13.5 × 4–5	3–6	10.5–13.5 × 3.5–4.5	Subglobose to ellipsoidal	Echinulate	4.5–5.5 × 4–5
*T.qii*	Biverticillate	4–6	7–11 × 2.5–3	2–4	7–9×2–2.5	Ovoid to subglobose	Echinulate	3–3.5
*T.thailandensis*	Biverticillate	3–5	7.2–10.9 × 2.2–3.4	3–7	11.5–13.7 × 1.5–2.4	Subglobose to ellipsoidal	Smooth	1.8–2.4 × 1.7 –2.3
